# Comparative genomic analysis of the R2R3 MYB secondary cell wall regulators of Arabidopsis, poplar, rice, maize, and switchgrass

**DOI:** 10.1186/1471-2229-14-135

**Published:** 2014-05-18

**Authors:** Kangmei Zhao, Laura E Bartley

**Affiliations:** 1 Department of Microbiology and Plant Biology, University of Oklahoma, Norman, OK 73019, USA

**Keywords:** Comparative genomics, Secondary cell wall, R2R3 MYB, Transcription factor, Homolog, Ortholog, Biofuel

## Abstract

**Background:**

R2R3 MYB proteins constitute one of the largest plant transcription factor clades and regulate diverse plant-specific processes. Several R2R3 MYB proteins act as regulators of secondary cell wall (SCW) biosynthesis in *Arabidopsis thaliana* (At), a dicotyledenous plant. Relatively few studies have examined SCW R2R3 MYB function in grasses, which may have diverged from dicots in terms of SCW regulatory mechanisms, as they have in cell wall composition and patterning. Understanding cell wall regulation is especially important for improving lignocellulosic bioenergy crops, such as switchgrass.

**Results:**

Here, we describe the results of applying phylogenic, OrthoMCL, and sequence identity analyses to classify the R2R3 MYB family proteins from the annotated proteomes of Arabidposis, poplar, rice, maize and the initial genome (v0.0) and translated transcriptome of switchgrass (*Panicum virgatum*). We find that the R2R3 MYB proteins of the five species fall into 48 subgroups, including three dicot-specific, six grass-specific, and two panicoid grass-expanded subgroups. We observe four classes of phylogenetic relationships within the subgroups of known SCW-regulating MYB proteins between Arabidopsis and rice, ranging from likely one-to-one orthology (for AtMYB26, AtMYB103, AtMYB69) to no homologs identifiable (for AtMYB75). Microarray data for putative switchgrass SCW MYBs indicate that many maintain similar expression patterns with the Arabidopsis SCW regulators. However, some of the switchgrass-expanded candidate SCW MYBs exhibit differences in gene expression patterns among paralogs, consistent with subfunctionalization. Furthermore, some switchgrass representatives of grass-expanded clades have gene expression patterns consistent with regulating SCW development.

**Conclusions:**

Our analysis suggests that no single comparative genomics tool is able to provide a complete picture of the R2R3 MYB protein family without leaving ambiguities, and establishing likely false-negative and -positive relationships, but that used together a relatively clear view emerges. Generally, we find that most R2R3 MYBs that regulate SCW in Arabidopsis are likely conserved in the grasses. This comparative analysis of the R2R3 MYB family will facilitate transfer of understanding of regulatory mechanisms among species and enable control of SCW biosynthesis in switchgrass toward improving its biomass quality.

## Background

MYB proteins form one of the largest transcription factor families in plants. They regulate diverse processes including development, secondary metabolism, and stress responses [1,2]. MYB proteins are typified by a conserved DNA binding domain consisting of up to four imperfect repeats (R) of 50 to 54 amino acids. Characterized by regularly spaced tryptophan residues, each repeat contains two α–helices that form a helix-turn-helix structure, and a third helix that binds the DNA major groove [2-4]. MYB proteins are classified based on the sequence and number of adjacent repeats, with R1, R2R3, 3R and 4R proteins having one, two, three, and four repeats, respectively [2,5-7]. MYB proteins with one or more divergent or partial R repeat are classified as MYB-like or MYB-related [8]. Two repeat domains, either covalently or non-covalently associated, appear to be necessary and sufficient for high-affinity DNA binding [9].

In plants, the MYB R2R3 proteins are by far the most abundant of the MYB classes. R2R3 MYBs likely evolved from progenitor 3R MYB proteins by losing the R1 repeat [10]. The family subsequently underwent a dramatic expansion after the origin of land plants but before the divergence of dicots and grasses [10-12]. The whole-genome complements of R2R3 MYB proteins has been investigated in several plant species, including Arabidopsis, rice (*Oryza sativa*), poplar (*Populus trichocarpa*), grapevine (*Vitis vinifera*), and maize (*Zea mays*), often with the goals of identifying orthologous groups and species-diverged clades [13-17]. The Arabidopsis genome encodes 126 R2R3 MYB proteins, most of which have been divided into 25 subgroups based on conserved motifs in the C-terminal protein regions [2,13]. More recently, thirteen additional subgroups, for a total of 37 groups (G), were proposed based on comparative analysis of the R2R3 MYBs of *Arabidopsis* and maize [17].

**Figure 1:**
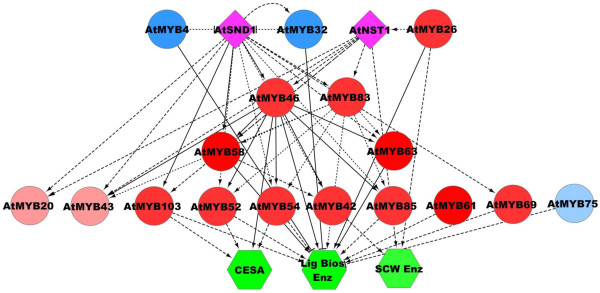
**Transcriptional regulation network of Arabidopsis known secondary cell wall R2R3 MYB proteins.** Pink and red symbols are positive regulators and blue are negative regulators. Nodes with darker shades show evidence of conservation in grasses that is absent for lighter shaded nodes (see text). MYBs are depicted by circles. Two crucial NAC-family transcriptional regulators, SND1, SECONDARY WALL-ASSOCIATED NAC DOMAIN PROTEIN1 and NST1, NAC SECONDARY WALL THINCKENING FACTOR 1, are depicted by diamonds. Other known regulators are excluded for simplicity [24,26]. Green hexagons represent genes that encode biosynthetic enzymes. Lig Bios Enz represents lignin biosynthesis enzymes, CESA is the cellulose synthases, and SCW Enz represents unspecified secondary cell wall synthesis enzymes. Solid edges represent direct interactions (i.e., evidence of physical promoter binding) and dashed edges represent indirect interactions (i.e., a change of gene expression with altered regulator expression). Indirect interactions may be direct, but not yet characterized. The figure was prepared with Cytoscape.

The function of R2R3 MYBs in regulating secondary cell wall (SCW) biosynthesis has garnered particular recent attention due to the importance of plant cell walls as a source of biomass for sustainable biofuel production [18,19]. Secondary walls form around many cell types after cessation of plant cell growth. Genetic studies have clearly demonstrated that thickened and chemically cross-linked SCWs function in structural support, water transport, and stress resistance [20]. SCWs are composed almost entirely of cellulose microfibrils encased by a network of (glucurano) arabinoxylan and phenylpropanoid-derived lignin. Studies mostly undertaken in Arabidopsis, a eudicot, have shown that numerous R2R3 MYBs are part of the complex regulatory network controlling formation of SCWs [21-25]. Figure 1 diagrams current understanding of the relationships among the 17 Arabidopsis R2R3 MYBs that have been identified so far to possibly function in SCW regulation. The network has multiple levels, though many higher-level regulators also directly regulate expression of genes encoding cell wall biosynthesis enzymes [22] (Figure 1). Table 1 summarizes the roles of individual Arabidopsis MYBs in SCW regulation and the initial forays into validating this regulatory network in grasses and poplar.

**Table 1:** Secondary cell wall (SCW)-associated R2R3 MYBs in dicots and grasses, organized based on phylogenetic tree topology

**Subgroup**	**Name**	**Function**	**Regulation and Phenotype**	**Reference**
G29	AtMYB26	Activator	Overexpression results in ectopic induction of SCW thickening and lignification.	[27]
G30	AtMYB103	Activator	Loss of function mutant reduces syringyl lignin; Overexpression increases SCW thickening in fibers; Regulates pollen development.	[28-30]
G21	AtMYB69	Activator	Dominant repression reduces SCW thickening in both interfascicular fibers and xylary fibers in stems.	[31]
G31	AtMYB46	Activator	Dominant repression reduces SCW thickening of fibers and vessels; Overexpression mutant leads to ectopic deposition of secondary walls.	[31-36]
G31	AtMYB83	Activator	Functionally redundant with AtMYB46; Overexpression induces ectopic SCW deposition.	[33,36]
G31	ZmMYB46	Activator	Overexpression in Arabidopsis induces ectopic deposition of lignin and xylan and an increases accumulation of cellulose in the walls of epidermis.	[37]
G31	OsMYB46	Activator	Overexpression in Arabidopsis induces ectopic deposition of lignin and xylan and an increases accumulation of cellulose in the walls of epidermis.	[37]
G31	PtrMYB20	Activator	Overexpression activates the biosynthetic pathway genes of cellulose, xylan and lignin.	[38]
G31	PtrMYB3	Activator	Overexpression activates the biosynthetic pathways genes of cellulose, xylan and lignin.	[38]
G8	AtMYB20	Activator	Activated by SND1 and NST1.	[31]
G8	AtMYB43	Activator	Activated by SND1 and NST1.	[31]
G8	AtMYB42	Activator	Activated by SND1 and NST1.	[31]
G8	AtMYB85	Activator	Overexpression results in ectopic deposition of lignin in epidermal and cortical cells in stems; Dominant repression reduces SCW thickening in both stem interfascicular fibers and xylary fibers.	[31]
G21	AtMYB52	Activator	Dominant repression reduces SCW thickening in both stem interfascicular fibers and xylary fibers.	[31]
G21	AtMYB54	Activator	Dominant repression reduced SCW thickening in both stem interfascicular fibers and xylary fibers.	[31]
G3.a	AtMYB58	Activator	Dominant repression reduces SCW thickening and lignin content; Overexpression causes ectopic lignification.	[30]
G3.a	AtMYB63	Activator	Dominant repression reduces SCW thickening and lignin content; Overexpression causes ectopic lignification.	[30]
G13.b	AtMYB61	Activator	Loss of function mutant reduces xylem vessels and lignification; Affects water and carbon allocation.	[39,40]
G4	AtMYB4	Repressor	Response to UV-B; Overexpression lines show white lesion in old leaves.	[41,42]
G4	AtMYB32	Repressor	Regulates pollen formation.	[42]
G4	ZmMYB31	Repressor	Overexpression reduces lignin content without changing composition.	[43]
G4	ZmMYB42	Repressor	Overexpression decreases S to G ratio of lignin.	[43,44]
G4	PvMYB4	Repressor	Overexpression represses lignin content.	[45]
G6	AtMYB75	Repressor	Represses lignin biosynthesis and cell wall thickening in xylary and interfascicular fibers.	[46]

Biomass from cereals and other grasses is of special interest as they constitute ~55% of the lignocellulosic material that can be sustainably produced in the U.S. [47]. Grass and eudicot SCWs have partially divergent compositions [24,48,49]. In addition, grasses and dicots have different patterns of vasculature, with its associated secondary wall, within leaves and stems. Grasses, as monocotoledenous plants, produce leaves with parallel venation; whereas, dicot leaf venation is palmate or pinnate. In grasses with C4 photosynthesis, including maize and switchgrass, there is further cell wall thickening of the bundle sheath cells to support the separate phases of photosynthesis. Within stems, vascular bundles of dicots form in rings from the cambium; whereas, grass stems, which lack a cambium layer, exhibit a scattered (e.g., atactostele) pattern [24,50,51]. Outside of the vasculature, the occurrence and patterning of extraxylary sclerenchyma cells, which are typified by thick cell walls, also varies between monocots and dicots [50]. Grasses have, for example, a sclerenchyma layer circumscribing their root cortex that is absent in Arabidopsis and other dicots [50,52].

We postulate that the differences in composition and patterning of grass SCWs may have resulted in gains or losses of regulatory modules in grasses relative to dicots. The phylogenetic analysis of two dicots and three grasses presented here aims to refine this hypothesis. By comparing the R2R3 MYBs across diverse species, our goal is to identify conserved or expanded protein groups that may regulate grass SCW synthesis. Furthermore, examining the entire R2R3 MYB family will facilitate study of MYB subgroups that regulate other important processes.

Our analysis is anchored on the relatively well-studied R2R3 MYBs of Arabidopsis [2], which is in the eurosid I clade of eudicots (family Brassicaceae). We have also analyzed the angiosperm tree species poplar, which is an important species from an ecological context, is now used by the pulp and paper industry, and is also an major potential source of biomass for lignocellulosic biofuels. Poplar is in the family Salicaceae, which lies within the eurosid II clade, and shared a common ancestor with Arabidopsis approximately 100 million years ago [53]. The poplar genome has been sequenced for several years [54] and an early version was analyzed for R2R3 MYB content [15]. To represent grasses, we have analyzed rice, maize, and switchgrass (*Panicum virgatum* L.). Rice is in the subfamily Erhardtoideae, whereas, maize and switchgrass are both in the Panicoideae [55]. Rice was the first grass to have its genome sequenced [56] and, among grasses, rice genomics and reverse genetic resources are arguably the best-developed [57]. As a staple for about half of the human population, rice is an extremely important crop; consequently, its straw represents ~23% of global agriculture waste for which one potential use is lignocellulosic biofuels [58]. Previous cataloging of rice R2R3 MYBs [14,16] had complementary foci to that presented here. Maize is also a very important food, feed, and first generation bioethanol crop with abundant genetic and genomic resources. Based on its recently sequenced genome [59], Du et al. conducted a phylogenetic analysis of its R2R3 MYBs similar to that here and serving, in part, as validation. Lastly, we have examined the R2R3 MYB complement of the large-stature, C4 perennial grass, switchgrass, which is currently used for forage and in erosion control, and is being actively and widely developed as a bioenergy crop [49,60-62]. The tetraploid (1n = 2×) genome size of lowlands and some upland switchgrass ecotypes is approximately 1.4 Mbp, which includes whole genome duplication approximately 1 million years ago [63]. Switchgrass is an outcrossing species. In part due to the heterozygosity of the genome, a psuedomolecule chromosomal assembly of the switchgrass genome was not available until recently (http://www.phytozome.net/panicumvirgatum) [62].

Comparisons between model species, with their relatively small genomes, and non-models are often made more challenging due to whole genome and localized duplication events. To facilitate such translational science, multiple approaches have been developed for comparing the gene complement and genomic arrangement of whole genomes or particular biologically and economically relevant protein families [64]. Commonly employed methods include phylogentic analysis based on sequence alignments (e.g., [15,17]), pair-wise quantitation of sequence identity (e.g., [65]), and more complex tools, like OrthoMCL (e.g., [66,67]). Such approaches vary in their sophistication, underlying assumptions, and the level of time, attention, and bioinformatics-acumen required. Another aim of this work is to analyze the apparent performance of commonly used tools at identifying individual genes for further study and manipulation.

Here, we present an investigation of the R2R3 MYB transcription factor family focusing on the non-model species switchgrass, using various comparative genomic approaches. We identified a total of 48 to 52 R2R3 MYB subgroups, most of which are common among all five species and similar to those previously described. Phylogenetic analysis reveals four patterns of conservation among proteins related to the known SCW R2R3 MYB regulators of Arabidopsis, ranging from one-to-one conservation between Arabidopsis and rice to unconserved between grasses and Arabidopsis, though most Arabidopsis SCW-regulating MYBs do appear to have orthologs in grasses. To clarify which proteins from paralogous groups are more likely to act as functional orthologs, we also applied sequence identity and OrthoMCL analysis to the R2R3 MYB protein sequences. Moreover, switchgrass gene expression data provide evidence that particular paralogs are more likely to function in SCW regulation and that some novel, grass-diverged MYB genes are expressed in tissues undergoing SCW formation, suggesting avenues for improvement of economically important traits.

## Results and discussion

### Identification of R2R3 MYB proteins

**Figure 2:**
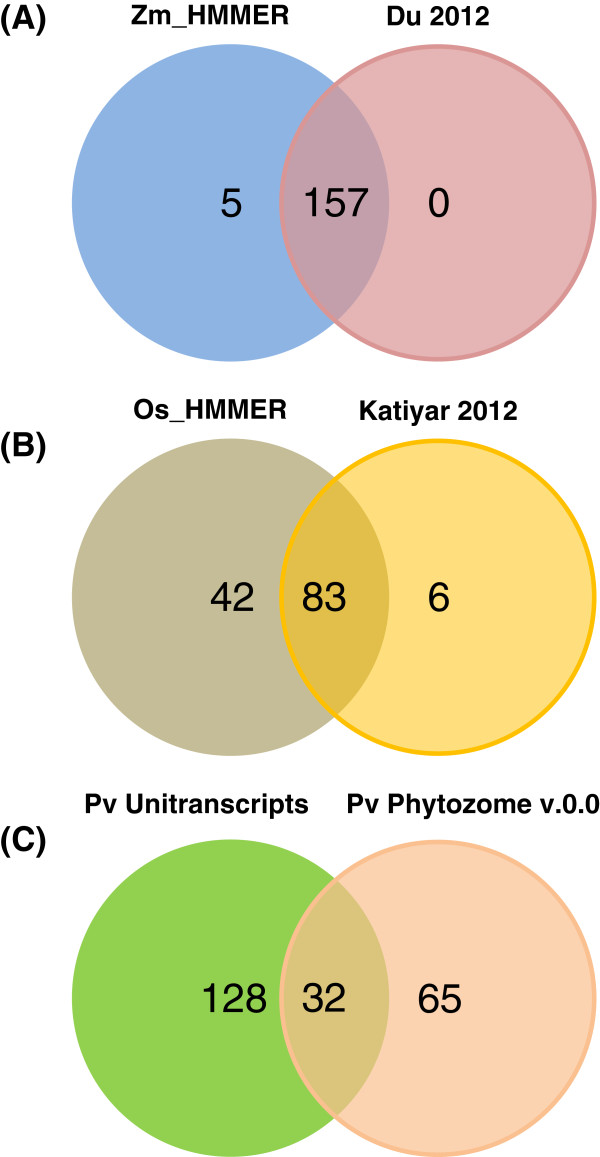
**Summary of this study compared to previous ones on R2R3 MYBs and the source of switchgrass sequences. (A)** Comparison of maize R2R3 MYB sequences identified here using HMMER and PROSITE prediction with previously published data from Du et al. 2012 [17]. **(B)** Comparison of rice R2R3 MYB sequences identified here using HMMER and PROSITE with published data from Katiyar et al. 2012 [16]. **(C)** Sources of switchgrass R2R3 MYB family sequences used in this analysis.

R2R3 MYB proteins regulate diverse plant-specific processes, including secondary cell wall synthesis, stress responses, and development. To identify the R2R3 MYBs in the annotated genomes of poplar, rice, and maize, we used a Hidden Markov Model built from the Arabidopsis R2R3 MYB proteins of Arabidopsis. We discarded identical sequences and loci that lack the two complete R2R3 repeats following manual inspection and PROSITE characterization. Table 2 summarizes the number of unique putative R2R3 MYBs that we found in the genomes of each species, which are listed in Additional file 1: Table S1. The species with smaller genomes, Arabidopsis and rice, possess similar numbers of R2R3 MYBs, whereas, organisms with larger genomes have greater numbers. Figure 2A and 2B show that our method may provide a more complete catalog of R2R3 MYBs in rice and maize compared with recently published analyses [16,17]. The six sequences that Katiyar *et al*. identified from rice that are excluded from our list lack the R2R3 repeats compared with the PROSITE profile. The previous analysis in maize relied on BLASTP, which may be slightly less sensitive to distantly related sequences [68]. For poplar, Wilkins et al. [15] identified 192 unique R2R3 MYBs, similar to the 202 that we were able to distinguish, and in keeping with the observation that poplar has undergone an enormous expansion in the number of R2R3 MYBs since its last common ancestor with Arabidopsis. The sequences used in the previous poplar analysis are not available, preventing a specific comparison with that work.

**Table 2:** R2R3 MYB proteins in analyzed species

**Clade**	**Organism**	**Sequence source**	**R2R3 MYBs**
Eudicot	Arabidopsis	TAIR v.10	126
	Poplar	Phytozome v.3	202
Grass	Rice	Rice Genome Annotation v.7	125
	Maize	Phytozome v.2	162
	Switchgrass	Phytozome v.0.0	230
		Switchgrass Functional Genomics Sever	

Arabidopsis R2R3 MYB protein sequences were identified previously [13].

For switchgrass, we combined the R2R3 MYBs that we identified from the annotated proteins in the DOE-JGI v0.0 genome with those from our translation of the unitranscript sequences available from the Switchgrass Functional Genomics Server. Figure 2C shows the distribution of the putative R2R3 MYBs from the two sources. Approximately twice as many proteins were identified from the translated unitranscripts than the v0.0 genome annotation. This is in part due to the fact that multiple genotypes were used to assemble the EST resource and about 10% of MYBs from the unitranscripts are attributed to the Kanlow cultivar. In addition, the presence of sequences within the genome that did not pass the protein annotation quality control (see Methods) may decrease the protein complement of the v0.0 genome. That we identified more putative R2R3 MYBs from switchgrass than the other species likely reflects the recent whole genome duplication of switchgrass [63], though the total may be inflated by the heterozygous nature of the outcrossed genotypes sequenced and include alleles or unaligned splice-variants.

### Comparative phylogenetic analysis of R2R3 MYB proteins in dicots and grasses

To examine broad conservation and divergence of R2R3 MYB proteins among the species examined, we inferred the phylogenetic relationships among the complete set of R2R3 MYB family proteins from Arabidopsis, poplar, rice, maize and switchgrass. We also accounted for the 25 published subgroups of Arabidopsis R2R3 MYB proteins and the more recently recognized 37 subgroups from a comparative analysis of R2R3 MYB family of Arabidopsis and maize [13,17]. Proteins clustered in each subgroup of the phylogenetic tree frequently possess similar functions. On the other hand, general functions, such as regulation of specialized metabolism, are not isolated to specific or closely related subgroups. For example, characterized Arabidopsis R2R3 MYBs that regulate plant cell wall biosynthesis are spread among the subgroups G (or S) 3, G4, G6, G8, G13, G21 G29, G30, and G31 (Table 1).

We find that R2R3 MYB proteins from the five species fall into approximately 48 subgroups (Table 3, Additional file 2: Figure S1), with G38 to G48 emerging as novel groups in the five-species phylogeny. In addition, four of the previously described subgroups, G3, G13, G14 and G17, are poorly supported in our analysis and we have further divided them into a and b subclades. We identified three dicot-specific groups (G6, 10, 15) and six grass-specific groups (G27, G32, G35, G43, G45, G46) plus G3.b. These non-conserved groups likely evolved after the divergence of eudicots and grasses 140 to 150 million years ago [10-12]. In addition, poplar possesses four unique subgroups (G38, G39, G40, G48). Previous analysis showed that whole genome duplication and R2R3 MYB-specific expansions contributed to the evolution of MYBs in poplar [15]. Though difficult to compare directly, Wilkins et al. did identify 6 subgroups in poplar that were not shared with Arabidopsis [15]. We also find continued support for an Arabidopsis-specific subgroup, G12, which regulates glucosinolate biosynthesis and metabolism [69,70].

**Table 3:** Subgroups of R2R3 MYB proteins from Arabidopsis (At), poplar (Ptr), rice (Os), maize (Zm) and switchgrass (Pv) defined by neighbor-joining phylogenetic reconstruction

**Sub-group**^**a**^	**Subgroup distribution**^**b**^	**Bootstrap score**	**At**	**Ptr**	**Os**	**Zm**	**Pv**	**Previous C-terminal motif identification**^**c**^	**Names of SCW regulators (AtMYB#)**
**G1**	Panicoid-Expanded^d^	66	5	4	7	12	14	I	0
**G2**	ND^e^	37	3	4	3	5	8	P	0
**G3.a**	ND^e^	3	4	2	1	1	2	P	58, 63
**G3.b**	Grass-Expanded	46	0	0	2	5	6	N	0
**G4**	ND^e^	14	6	7	8	10	22	I	5
**G5**	ND^e^	13	1	9	2	2	1	N	0
**G6**	Dicot-Expanded	7	4	8	0	0	0	I	75
**G7**	ND^e^	17	2	1	2	5	4	N	0
**G8**	ND^e^	89	4	6	5	8	17	P	20, 43, 42, 85
**G9**	ND^e^	51	2	4	3	4	7	P	0
**G10**	Dicot-Expanded	100	2	3	0	0	0	P	0
**G11**	ND^e^	92	4	6	1	2	0	I	0
**G12**	Arabidopsis-Specific	26	6	0	0	0	0	I	0
**G13.a**	ND^e^	21	1	2	1	2	5	P	0
**G13.b**	ND^e^	5	4	7	5	7	10	P	61
**G14.a**	ND^e^	33	2	5	2	2	1	N	0
**G14.b**	ND^e^	43	6	8	8	11	22	N	0
**G15**	Dicot-Expanded	39	4	5	0	0	0	I	0
**G16**	ND^e^	30	3	2	3	3	8	P	0
**G17.a**	ND^e^	93	2	2	3	5	4	N	0
**G17.b**	ND^e^	86	3	5	3	4	3	P	0
**G18**	ND^e^	7	7	5	2	2	3	N	0
**G19**	ND^e^	59	3	2	1	0	0	N	0
**G20**	Panicoid-Expansion	88	6	8	5	13	10	I	0
**G21**	ND^e^	20	8	13	5	8	14	I	52, 54, 69
**G22**	ND^e^	62	4	6	3	5	12	P	0
**G23**	ND^e^	98	3	1	1	1	2	N	0
**G24**	ND^e^	88	3	4	3	3	5	I	0
**G25**	ND^e^	29	7	6	5	4	8	I	0
**G26**	ND^e^	80	1	4	2	3	0	N	0
**G27**	Grass-Expanded	63	0	0	2	3	1	N	0
**G28**	ND^e^	25	1	7	1	1	0	N	0
**G29**	ND^e^	40	2	5	2	2	3	N	26
**G30**	ND^e^	100	1	2	1	1	2	N	103
**G31**	ND^e^	99	2	4	1	1	2	N	46, 83
**G32**	Grass-Expanded	100	0	0	1	5	1	N	0
**G33**	ND^e^	100	1	3	1	3	4	N	0
**G34**	ND^e^	100	1	3	0	1	0	N	0
**G35**	Grass-Expanded	42	0	0	2	4	6	N	0
**G36**	ND^e^	25	0	2	2	2	3	N	0
**G37**	ND^e^	100	2	2	1	1	1	N	0
**G38**	Poplar-Specific	13	0	7	0	0	0	N	0
**G39**	Poplar-Specific	86	0	3	0	0	0	N	0
**G40**	Poplar-Specific	100	0	4	0	0	0	N	0
**G41**	ND^e^	100	1	5	7	3	0	N	0
**G42**	ND^e^	100	0	0	1	0	3	N	0
**G43**	Grass-Expanded	75	0	0	2	1	5	N	0
**G44**	Rice-Specific	99	0	0	7	0	0	N	0
**G45**	Grass-Expanded	100	0	0	1	1	3	N	0
**G46**	Grass-Expanded	97	0	0	2	3	7	N	0
**G47**		21	0	5	0	1	0	N	0
**G48**	Poplar Specific	37	0	4	0	0	0	N	0

Assignment to a subgroup is based on the 5-species neighbor-joining tree with 500 bootstraps (Additional file 2: Figure S1).

^b^Distribution or expression of each subgroup in the clades examined.

^c^C-terminal conserved motifs were analyzed using MEME for each subgroup and compared to known motifs present in the 25 subgroups of Arabidopsis R2R3 MYB family. I: Previously identified; P: Partially previously identified; N: Not previously identified. The last column lists the Arabidopsis (At) secondary cell wall (SCW) regulators by their numeric names.

^d^Panicoid-expanded refers to the pattern in maize and switchgrass.

^e^ND indicates that no subgroup distribution pattern was detected.

With MEME, we found that many of the subgroups designated in our analysis possess conserved C-terminal motifs, often supporting and extending those initially identified in the Arabidopsis R2R3 MYB subgroups (Table 3, Additional file 3: Table S2) [13]. Located downstream of the N-terminal MYB DNA-binding domains, C-terminal motifs have been hypothesized to contribute to the biological functions of R2R3 MYB proteins [2,13]. For example, the C-terminal motif, LNL [ED] L, of AtMYB4, found to be conserved in the analysis presented here, is required for repression of the transcription at target promoters (Additional file 3: Table S2) [41]. The large number of sequences in our analysis apparently improved our sensitivity allowing identification of many motifs that were not apparent previously, including those of subgroup G23, and candidate motifs within the new subgroups (Additional file 3: Table S2). Of the 25 original R2R3 MYB family subgroups of Arabidopsis [13], we found that all but 7 (G3.b, G5, G14.a and G14.b, G17.a, G18, G19 and G22) contain the same or similar motifs as identified previously in the corresponding Arabidopsis subgroups (Table 3, Additional file 3: Table S2). Differences in identified motifs may stem from uncertainties in the subgroup designations. For the subgroups with different conserved motifs, two of them, G19 and G22, have bootstrap values higher than 50 in the five species phylogenetic tree; whereas, the phylogenies of subgroups G5 and G18, are poorly supported. The subdivided subgroups had variable effects on the identified motifs. Subgroups G3.a (but not G3.b) and G17.b (but not G17.a) possess the previously identified motifs. Both subgroups G13.a and .b contain the previously identified motif. In contrast, the original motif is not identifiable in either G14.a or .b.

### Identification of putative orthologs of Arabidopsis SCW MYB across different species

**Figure 3:**
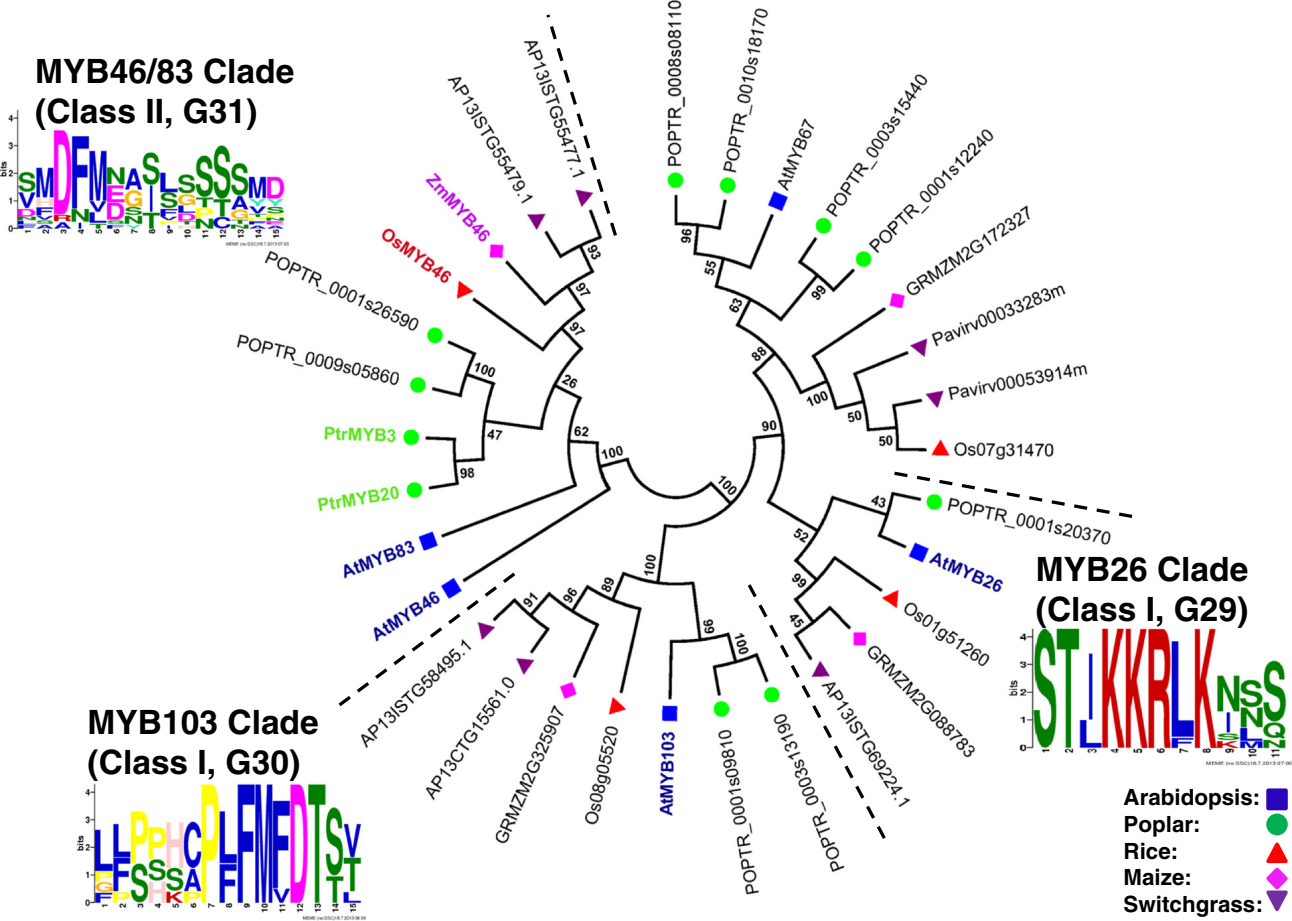
**Maximum likelihood phylogenetic analysis of subgroups G29, G30, and G31 suggests that the function of the secondary cell wall (SCW) regulators, MYB46, MYB83, MYB103, and MYB26, are conserved between grasses and Arabidopsis.** Poplar and switchgrass show gene duplication in the MYB46/83 and MYB103 clades. MYB proteins represented with bold and colored text are characterized SCW regulators in each species. Support values are from 1000 bootstrap analyses. Each logo is the C-terminal conserved motifs with the lowest E-value identified for the subgroup.

**Figure 4:**
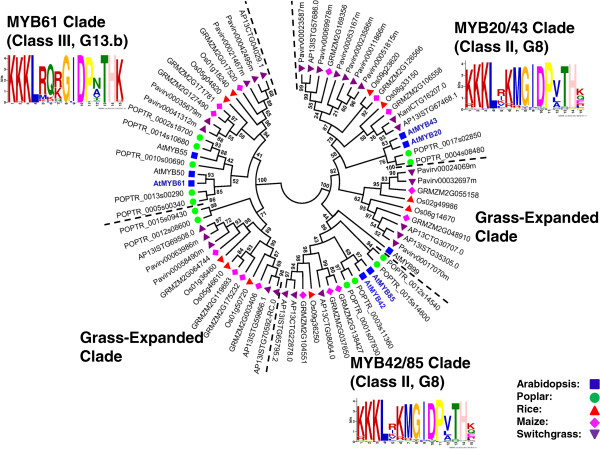
**Maximum likelihood phylogenetic analysis of subgroups G8 and G13.b suggests gene duplication in dicots and grasses after divergence.** MYB42/85 and MYB20/43 clades show expansion in maize and switchgrass. Two grass-expanded clades are indicated. MYB proteins shown with bold and colored text are characterized SCW regulators. Support values are from 1000 bootstrap analyses. Each logo is the C-terminal conserved motifs with the lowest E-value identified for the subgroup.

**Figure 5:**
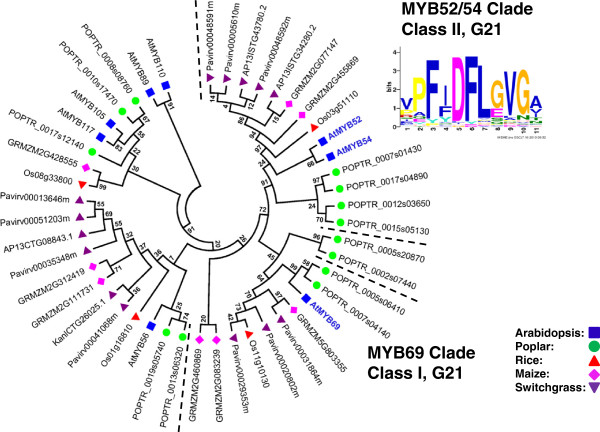
**Maximum likelihood phylogenetic analysis of subgroup G21 suggests orthologous and paralogous relationships in dicots and grasses.** The MYB69 clade is conserved across evolution with putative orthologs in the five species. The MYB52/54 clade shows expansion in poplar and switchgrass. MYB proteins shown with bold and colored text are characterized SCW regulators. Support values are from 1000 bootstrap analyses. Each logo is the C-terminal conserved motifs with the lowest E-value identified for the subgroup.

**Figure 6:**
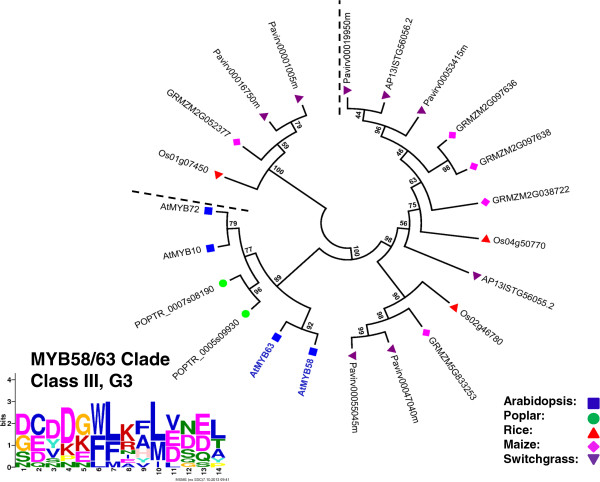
**Maximum likelihood phylogenetic analysis of subgroup G3.a and G3.b suggests that MYB58/63 clade underwent expansion after the divergence of dicots and grasses.** AtMYB10 and AtMYB72 are involved in cesium toxicity and pathogen resistance, which indicates neofunctionalization after duplication. MYB proteins shown with bold and colored text are characterized SCW regulators. Support values are from 1000 bootstrap analyses. Each logo is the C-terminal conserved motifs with the lowest E-value identified for the subgroup.

**Figure 7:**
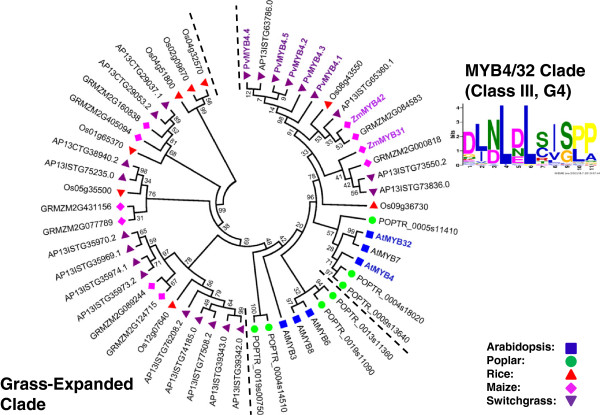
**Maximum likelihood phylogenetic analysis of subgroup G4 suggests expansion of this group in both grasses and dicots since the last common ancestor.** The MYB4/32 clade has many paralogs in dicots; however ZmMYB32, ZmMYB41 and PvMYB4.a have been shown to have function similarly to AtMYB4 and AtMYB32. PvMYB4.a to e are likely alleles among each other based on protein sequence similarity. MYB proteins shown with bold and colored text are characterized SCW regulators. Support values are from 1000 bootstrap analyses. Each logo is the C-terminal conserved motifs with the lowest E-value identified for the subgroup.

**Figure 8:**
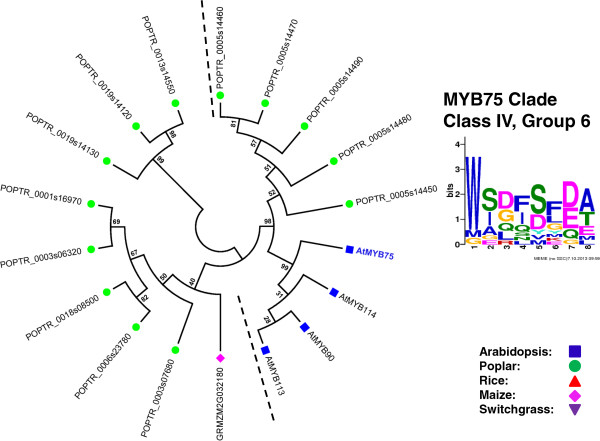
**Maximum likelihood phylogenetic analysis of subgroups G6 and G47 suggests that AtMYB75 is a dicot-specific SCW repressor without homologs in grasses.** MYB proteins shown with bold and colored text are characterized SCW regulators. Support values are from 1000 bootstrap analyses. Each logo is the C-terminal conserved motifs with the lowest E-value identified for the subgroup.

To identify the putative SCW-associated R2R3 MYB proteins from each species, we performed a more focused analysis of the subgroups containing the known Arabidopsis SCW MYBs. For this, we identified related proteins from the multi-species neighbor-joining tree (as corroborated by dual Arabidopsis-other species trees), grouped closely related subgroups together, realigned these sequences, and inferred maximum likelihood phylogenies. The results are summarized in Figures 3, 4, 5, 6, 7 and 8 and Table 4. We have sorted the R2R3 SCW MYB clades into four classes by comparing the relationships between the proteins of Arabidopsis and rice—the species with the smallest genomes examined here. The classes are as follows: one-to-one relationships (class I), duplication in Arabidopsis and both of them are SCW regulators (class II), expansion in Arabidopsis with non-SCW R2R3 MYBs (class III), and no orthologs identifiable in the grasses examined (class IV). In addition to the in-depth phylogenetic analysis, we used OrthoMCL and sequence identity as alternatives for identifying orthologous groups of R2R3 MYB proteins from the five species. OrthoMCL groups putative orthologs and paralogs based on BLAST scores across and within species and then resolves the many-to-many orthologous relationships using a Markov Cluster algorithm [71]. We analyzed sequence identity using alignments built with MUSCLE, which combines progressive alignment and iterative refinement [72]. Table 4 summarizes the results of all of these analyses.

**Table 4:** Groups of homologous proteins from poplar, rice, maize and switchgrass relative to the Arabidopsis R2R3 MYB secondary cell wall (SCW) regulators

**Class**	**Arabidopsis**	**Poplar POPTR_00**	**Sequence identity (%)**	**Rice LOC_Os**	**Sequence identity (%)**	**Maize GRMZM**	**Sequence identity (%)**	**Switchgrass**	**Sequence identity (%)**
**I**	AtMYB26	01s20370	47	01g51260	45	2G0887834	45	AP13ISTG69224	44
**I**	**AtMYB103**	**03s13190**	**60**	**08g05520**	**50**	**2G325907**	**48**	**AP13CTG15561**	**51**
		01s09810	62					**AP13ISTG58495**	**50**
**I**	**AtMYB69**	**07s04140**	**53**	11g10130	47	**5G803355**	**48**	**Pavirv00031864m**	**50**
				05s06410	53			Pavirv00029353m	50
								Pavirv00020802m	49
**II**	AtMYB46^a^	PtrMYB3	58^b^	**OsMYB46**	**47**^**a**^	**ZmMYB46**	**49**^**a**^	**AP13ISTG55479**	**50**^**a**^
	AtMYB83^b^	PtrMYB20	57^b^					**AP13ISTG55477**	**51**^**b**^
		09s05860	53^a^						
		01s26590	54^a^						
**II**	**AtMYB20**^**a**^	04s08480	58^b^	09g23620	54^a^	2G169356	**55**^**a**^	**Pavirv00023586m**	**69**^**a**^
	***AtMYB43***^***b***^	***17s02850***	***58***^***a***^	***08g33150***	***56***^***a***^	2G126566	**52**^**a**^	***KanlCTG16207***	***53***^***a***^
								***AP13ISTG67468***	***51***^***a***^
								Pavirv00053167m	60^a^
								AP13ISTG57686	56^a^
								Pavirv00069978m	56^a^
								Pavirv00023587m	53^a^
								Pavirv00051815m	57^a^
								Pavirv00011866m	57^a^
**II**	**AtMYB42**^**a**^	**03s11360**	**61**^**b**^	**09g36250**	**51**^**a**^	**2G104551**	**52**^**b**^	**AP13ISTG65795**	**52**^**b**^
	***AtMYB85***^***b***^	01s07830	61^a^			2G138427	53^b^	**AP13CTG22878**	**52**^**b**^
		15s14600	55^b^			2G037650	52^b^	AP13CTG08064	53^b^
		12s14540	57^b^						
**II**	**AtMYB52**^**a**^	**17s04890**	**55**^**a**^	**03g51110**	**52**^**a**^	**2G455869**	**53**^**a**^	**AP13ISTG34280**^**d**^	**59**^**a**^
	**AtMYB54**^**b**^	15s05130	57^b^			2G077147	52^a^	**AP13ISTG43780**^**d**^	**54**^**b**^
		12s03650	58^b^					**Pavirv00048592m**^**d**^	**54**^**b**^
		07s01430	53^a^					**Pavirv00048591m**^**d**^	**55**^**b**^
								Pavirv00005610m	52^b^
**III**	**AtMYB58**^**a**^	**07s08190**	**48**^**a**^	**02g46780**	**49**^**a**^	**5G833253**	**46**^**a**^	**Pavirv00055045m**	**47**^**b**^
	**AtMYB63**^**b**^	05s09930	48^a^	04g50770	48^a^	2G097636	47^a^	AP13ISTG56055	38^a^
						2G097638	50^a^	Pavirv00019950m	49^a^
						2G038722	47^a^	Pavirv00047040m	51^b^
								AP13ISTG56056	49^a^
								Pavirv00053415m	50^a^
**III**	**AtMYB61**^**a**^	**05s00340**	**53**^**a**^	**05g04820**	**57**^**b**^	**2G127490**	**56**^**b**^	**AP13CTG04029**	**56**^**b**^
	AtMYB50^b^	**13s00290**	**60**^**a**^	**01g18240**	**57**^**b**^	**2G171781**	**56**^**b**^	**Pavirv00042495m**	**56**^**b**^
	AtMYB55^c^	02s18700	56^c^			**2G017520**	**56**^**b**^	**Pavirv00021467m**	**56**^**b**^
		14s10680	57^c^					**Pavirv00035679m**	**58**^**b**^
								**Pavirv00041312m**	**58**^**b**^
**III**	**AtMYB4**^**a**^	**05s11410**	**67**^**a**^	**09g36730**	**68**^**a**^	**2G000818**	**75**^**a**^	**AP13ISTG73550**	**68**^**a**^
	***AtMYB32***^***b***^	09s13640	66^a^	08g43550	56^b^	**ZmMYB31**	**65**^**a**^	**AP13ISTG73836**	**70**^**a**^
		04s18020	70^a^			***ZmMYB42***	***65***^***a***^	**PvMYB4.a**^**d**^	**64**^**a**^
						**(2G084583)**	**66**^**a**^	**(PvMYB4.b**^**d**^**)**	**64**^**a**^
								**(PvMYB4.c**^**d**^**)**	**64**^**a**^
								**(PvMYB4.d**^**d**^**)**	**64**^**a**^
								**(PvMYB4.e**^**d**^**)**	**64**^**a**^
**IV**	**AtMYB75**	**05s14450**	**67**^**a**^						
	**AtMYB90**	**05s14460**	**67**^**a**^						
	**AtMYB113**	**05s14470**	**67**^**a**^						
	**AtMYB114**^**a**^	**05s14480**	**70**^**a**^						
		**05s14490**	**72**^**a**^						

Classes refer to the phylogentic relationships between the Arabidopsis and rice proteins in the clade as described in the text. Proteins are divided within each class based on maximum likelihood phylogenic reconstruction. Bold font indicates putative orthologous and paralogous relationships based on OrthoMCL analysis. Italic and round brackets indicate additional OrthoMCL groups within the clade.

^a, b, c^Indicate proteins with highest sequence identity to the indicated Arabidopsis MYB.

^d^MYBs that have ≥99% protein sequence similarity that are likely allelic to each other.

^e^Arabidopsis MYBs implicated in functions besides SCW regulation with higher sequence identity to proteins from the other species compared with the At SCW MYBs in the same clade.

**Figure 9:**
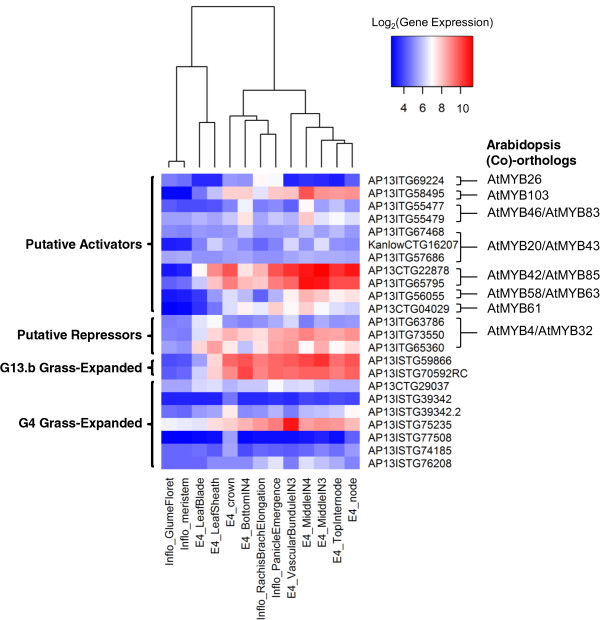
**Gene expression analysis of switchgrass MYBs that are putative SCW-related activators or repressors and members of grass-expanded clades.** The heatmap represents the log_2_ of the expression data, which are normalized mean values of three biological replicates in the same experiments from the Switchgrass Functional Genomics Server (http://switchgrassgenomics.noble.org/). The blue indicates lower expression and red, higher expression. The relationships among columns are based on hierarchical clustering. The orthologs/co-orthologs from Arabidopsis are listed. Among the repressors with gene expression available, PvMYB4.d_AP13ITG63786 is one of the published homologs of AtMYB4/32 in switchgrass and it has 100% sequence similarity with PbMYB4.d with low expression in most of the tissues. The labels of tissues and developmental stages are abbreviated using the following scheme: from the inflorescence (Inflo) the meristem, glume floret, rachis branch during elongation, and panicle during emergence; from the tiller at elongation stage 4 (E4) the crown, leaf blade, leaf sheath, and stem the stem segments as follows: nodes, top internode, middle of internode (IN) 3, vascular bundle of IN 3, middle of IN 4, and the bottom of IN 4 [73].

To gain further support for our tentative identification of switchgrass SCW R2R3 MYBs, we examined their patterns of expression, as available, using the switchgrass gene expression atlas [73]. Of particular relevance, that study included gene expression of internode 4 of tillers at elongation stage 4, which is informative for the investigation of secondary development and recalcitrance in stem tissues (Figure 9) [52], Saha, in prep].

### Class I: One-to-one relationships

Proteins in Class I show one-to-one conservation among Arabidopsis, rice, and maize and relatively modest expansion in poplar and switchgrass compared with other classes. The group consists of AtMYB26, AtMYB103 and AtMYB69 (Figures 3 and 5). For these and other classes, it remains a formal possibility that duplication and gene loss have occurred in other species relative to Arabidopsis resulting in pseudo-orthologs [74]. However, for the proteins in Class I, the expression patterns of the putative switchgrass orthologs support the hypothesis of conservation of function.

The only SCW MYB protein group with evidence of one-to-one conservation without duplication among all five species are those related to AtMYB26, which is also called *MALE STERILE35* (*MS35*). AtMYB26 was unclassified in the original subgroup analysis [13] and is a member of the small subgroup, G29 [17]. AtMYB26 is a high-level activator of SCW thickening in anthers, functioning in the critical process of pollen dehiscence [27]. Ectopic expression of A*tMYB26* upregulates *NST1* and *NST2* and causes SCW thickening, especially in epidermal tissues [27]. We found one putative ortholog of AtMYB26 in each species, suggesting that the critical function of MYB26 in reproduction may be conserved across evolution (Figure 3). Consistent with this, *AP13ISTG69224*, the putative switchgrass ortholog of *AtMYB26*, is lowly expressed in the stems (i.e., node and internode samples) and leaves at the E4 (elongation 4) stage, but more highly expressed in the inflorescence (Figure 9). The absence of duplication in switchgrass is unexpected given its recent genome duplication and likely reflects the incomplete genome sequence. On the other hand, sequence identity between AtMYB26 and its putative orthologs in grasses is relatively low, ~45%. Possibly due to that fact, OrthoMCL analysis did not identify AtMYB26 orthologs (Table 4). This amount of variation is consistent with divergence within this clade since the last common ancestor and sheds some doubt on the supposition of conservation of function in the absence of experimentation.

The other two clades included in Class I are those of AtMYB103 and AtMYB69, from subgroups G30 and G21, respectively. In Arabidopsis, these proteins are lower-level SCW activators, regulated by At*SND1* (Figure 1) [31]. *AtMYB103* is mainly expressed in the stem, where cells are undergoing secondary wall thickening [31]. *AP13ISTG58495* also has high expression levels in the vascular bundle and internodes (Figure 9). Thus, both phylogentic analysis and gene expression are consistent with maintenance of the function of these proteins across grasses and eudicots. Sequence identity between AtMYB103 and the putative grass orthologs is intermediate, ranging from 48% to 51%, and OrthoMCL mostly supports the phylogenetic analysis, further evidence that *AP13ISTG58495* may be a SCW regulator in switchgrass (Table 4). In rice, a preliminary study reported that RNAi lines of *OsMYB103* show a severe dwarf phenotype and did not grow to maturity [75]; whereas, only altered tapetum, pollen and trichome morphology were observed in Arabidopsis *AtMYB103* silencing mutants [28,29]. This difference in phenotypes caused by expression disruption of apparently orthologous genes between rice and Arabidopsis suggests differences in the SCW regulatory network between grasses and dicots not obvious from the phylogenetic relationships of the Class I proteins. For *AtMYB69*, of the three putative switchgrass co-orthologs, OrthoMCL identifies only *Pavirv00031864m* as an ortholog. These two proteins have 50% pairwise sequence identity and are similarly related to two other proteins in switchgrass (Table 4). No gene expression data for the three switchgrass co-orthologs are available to help resolve the question of whether there may be subfunctionalization in this family in switchgrass.

### Class II: SCW related co-orthologs in Arabidopsis

R2R3 MYB proteins in Class II underwent duplication in the Arabidopsis lineage, though the duplicates have apparently retained roles in regulating SCW biosynthesis. This class consists of AtMYB46 and AtMYB83, AtMYB42 and AtMYB85, AtMYB52 and AtMYB54, and AtMYB20 and AtMYB43.

AtMYB46 and AtMYB83, from subgroup G31, function redundantly to activate SCW biosynthesis [36]. AtMYB46 directly activates several genes related to cell wall synthesis and regulation, including *CESA*s, *AtMYB58*, *AtMYB63* and *AtMYB43* (Figure 1) [32,33]. Dominant repression of AtMYB46 reduces SCW accumulation, and simultaneous RNA interference of *AtMYB*46 and *AtMYB*83 deforms vessel and fibers [34,36]. Figure 3 shows the maximum likelihood phylogeny for these and this group provides evidence that it is part of a well-supported clade of likely co-orthologs. Consistent with this, functional data on the named poplar proteins and the rice and maize co-orthologs show that these proteins phenocopy *AtMYB46* and *AtMYB83* when heterologously expressed in Arabidopsis [37,38]. We found two putative co-orthologs of *AtMYB46* and *AtMYB83* in switchgrass, *AP13ISTG55479* and *AP13ISTG55477*, which are likely regulators of SCW biosynthesis (Figure 3). *AtMYB46* and *AtMYB83* are predominantly expressed at the sites of SCW synthesis—interfascicular fibers, xylary fibers, and vessels [32,34-36]. *AP13ISTG55479* and *AP13ISTG55477* also show relatively high expression in stems (Figure 9), with *AP13ISTG55477* being the more highly expressed of the two. OrthoMCL supports the orthologous relationship of grass MYB46-like proteins; however, the dicot sequences of the MYB46 clade do not cluster with those of the grasses, possibly due to the somewhat low sequence identity (47% to 50%; Table 4).

The other three Class II R2R3 MYB protein pairs are AtMYB42 and AtMYB85, and AtMYB20 and AtMYB43, from subgroup G8 (Figure 4); and AtMYB52 and AtMYB54 from subgroup G21 (Figure 5). These genes are expressed mainly in stems and specifically, in tested cases, in fiber and xylem cells and downregulated in a line silenced for *AtSND1* and *AtNST1 *[31]. Overexpression of *AtMYB85, AtMYB52*, or *AtMYB54* (but not of *AtMYB42, AtMYB20*, or *AtMYB43*) leads to ectopic deposition of lignin in epidermal and cortical cells in stems [31]. Moreover, RNAi of *OsMYB42/85* (*LOC_Os09g36250*) causes a severe dwarf phenotype [75]. The maximum likelihood phylogenic trees of each of these Arabidopsis protein pairs contains one or two rice proteins, one to three maize proteins and two or more poplar proteins (Figure 4, Figure 5, Table 4). The OrthoMCL result for AtMYB42, AtMYB85, AtMYB52 and AtMYB54 largely supports the phylogenetic topology, though excludes paralogs from poplar and maize (Table 4). OrthoMCL analysis separates AtMYB20 and AtMYB43 into different groups and identifies proteins in switchgrass as (co-) orthologs for each of these (Table 4). Among the switchgrass genes in Class II, *AP13CTG22878* and *AP13ISTG65795*, co-orthologs of *AtMYB42* and *AtMYB85*, are also highly expressed in stems, consistent with conservation of function in SCW regulation and providing no evidence of subfunctionalization (Figure 9). In contrast, co-orthologs of *AtMYB20* and *AtMYB43*, namely *AP13ISTG67468*, *KanlCTG16207* and *AP13ISTG57686*, are all expressed at low levels. No expression data are available for the switchgrass genes encoding AtMYB52 and AtMYB54 co-orthologs, four out of five of which may be putative alleles of each other due to high sequence identity (>99%; Table 4). In sum, though much of the phylogenic data are consistent with conserved function of other Class II proteins, for the three co-orthologs of AtMYB20 and AtMYB43, as well as the initial Arabidopsis genetic data, call into question the function of these proteins in SCW regulation.

### Class III: Non-SCW related paralogs in Arabidopsis

In Class III, the known Arabidopsis SCW regulators are closely related with other Arabidopsis R2R3 MYB proteins functioning in different biological processes. Thus, from phylogenetic analysis alone, it is difficult to hypothesize about the likely function of orthologs from other species. In this case, the amino acid identity within each clade and relationships identified by OrthoMCL aid in identification of likely functional orthologs [76]. Class III consists of AtMYB58 and AtMYB63, AtMYB61, and AtMYB4 and AtMYB32 (Figures 4, 6 and 7).

Functioning as lignin specific activators, *AtMYB58* and *AtMYB63* are regulated by *AtSND1* and its homologs, *AtNST1*, *AtNST2*, *AtVND6*, and *AtVND7*, and their target, *AtMYB46* (Figure 1) [77]. As shown in Figure 6, AtMYB58 and AtMYB63 are in subgroup G3 and are paralogous with AtMYB10 and AtMYB72, which are involved in cesium toxicity tolerance and beneficial bacteria responses, respectively [78,79]. This appears to be a case of neofunctionalization after gene duplication in the dicot lineage. Based on sequence similarity (Table 4), among the Arabidopsis proteins, AtMYB58 shares the highest similarity with those from other species; consistent with it being closest to the ancestral sequence and at least one homolog in other species having retained its function. *AtMYB58* and *AtMYB63* are predominantly expressed in vessels and fibers in Arabidopsis [77]. In contrast, their paralogs, *AtMYB10* and *AtMYB72* are mainly expressed in the inflorescence [14]. The switchgrass ortholog in this clade with gene expression data available, *AP13ISTG56055*, shows high expression in E4 vascular bundles and internodes, consistent with the possibility that they regulate SCW biosynthesis (Figure 9). Overexpression of the two *OsMYB58/63* genes was recently found to promote lignin deposition in rice stems, supporting their orthologous relationship with the AtMYB58 and 63 [75]. In the OrthoMCL analysis, AtMYB58 and AtMYB63 are paralogs and putative co-orthologs are found in the grasses. However, many related grass and poplar sequences are excluded from the orthologous relationship by OrthoMCL, possibly due to the somewhat low sequence identity (38% to 51%).

AtMYB61 is a SCW biosynthesis activator in subgroup G13.b that also belongs to Class III. AtMYB61 regulates water and sugar allocation and is mainly expressed in sink tissues. Loss-of-function mutants reduce xylem vessel formation and lignification [39]. AtMYB61 is closely related to AtMYB50 and AtMYB55 (Figure 4). The function of AtMYB50, with 66% identity to AtMYB61, has not been studied in detail to our knowledge. Its transcript is upregulated during geminivirus infection [80]. Another paralog, *AtMYB55*, is involved in leaf development [81]. We found that this clade is expanded in poplar and switchgrass; whereas, rice and maize possess two paralogs (Figure 4). RNAi of the two *OsMYB61*s downregulates the expression of *OsCAD2*, which encodes a lignin biosynthesis enzyme [75]. *AtMYB61* is expressed in xylem, leaf and root. In contrast, *AtMYB50* and *AtMYB55* are broadly expressed in Arabidopsis [8,39]. The ortholog in switchgrass for which expression data are available, *AP13CTG04029*, also shows high expression in the stem (Figure 9). Based on this expression pattern, we conclude that AP13CTG04029 may regulate SCW formation. Despite these functional and expression results, from sequence identity analysis alone, AtMYB50 appears to be most similar to the ancestral sequence, with the co-orthologs from Arabidopsis and the other species ranging in identity with it from 53% to 58%. On the other hand, OthoMCL analysis groups all of the grass co-orthologs and two from poplar with AtMYB61 (Table 4).

The last pair of proteins in class III is AtMYB4 and AtMYB32, which negatively regulate SCW biosynthesis (Figures 1 and 7). AtMYB4 is a repressor of lignin biosynthesis and ultraviolet B light responses [41]. AtMYB4 has two paralogs, AtMYB32 and AtMYB7, which repress Arabidopsis pollen cell wall development and are downregulated under drought stress, respectively [41,42,82]. In grasses, ZmMYB31, ZmMYB42 and PvMYB4a are all characterized orthologs of AtMYB4, that function as SCW biosynthesis repressors with somewhat paradoxically high expression in vascular tissues [43-45]. The characterized PvMYB4a is closely related to four other predicted proteins with amino acid identity >99%, which are putative alleles or splice variants of each other [45]. Among switchgrass ESTs, we found two additional orthologs of AtMYB4 that show high expression in vascular bundles, nodes, and internodes; whereas, the previously identified *PvMYB4d* is relatively lowly expressed (Figure 9). This difference in expression is consistent with subfunctionalization or loss of function of PvMYB4d after gene duplication in switchgrass. Data for the other PvMYB4 alleles are lacking. Consistent with their gene expression conservation, AtMYB4 is the most similar to the ancestral sequence, with orthologs from other species ranging in identity from 64% to 70% (Table 4). The MYB4/32 clade is disjointed in the OrthoMCL analysis. Most grass orthologs group with AtMYB4; however, ZmMYB42 and PvMYB4 cluster into two independent groups (Table 4).

### Class IV: No clear homologs in grasses

AtMYB75 is the only SCW R2R3 MYB protein in Class IV, for which we found no evidence of orthologs in grasses. AtMYB75 functions as a repressor of SCW biosynthesis and is also known as *PRODUCTION OF ANTHOCYANIN PIGMENT*1 (*PAP*1), with a role in positively regulating anthocyanin metabolism [21,46,83]. AtMYB75 belongs to the dicot-specific subgroup, G6, which includes AtMYB90, AtMYB113 and AtMYB114 (Table 2, Figure 8). Even when the relatively closely related G47 clade is included, our analysis separates AtMYB75 and the other members of G6 from all grass sequences. Among the G6 members, AtMYB114, which functions in nitrogen response, appears to be the most similar to the ancestral sequence, with the identity of co-orthologs from Arabidopsis and poplar with identity ranging from 67% to 72% (Table 4) [84-86]. Thus, AtMYB75 may have resulted from gene duplication in the Arabidopsis lineage and is likely a dicot-specific SCW repressor. OrthoMCL analysis supports the phylogenetic topology and only identifies putative AtMYB75 co-orthologs from poplar (Table 4).

### Expression of grass-expanded clades

In addition to putative (co-) orthologs of known SCW R2R3 MYBs, we noted the presence of grass-expanded clades in several of the subgroups that we examined in greater detail. As with the Class II proteins, these may have retained functions in SCW regulation or, as with Class III Arabidopsis proteins, developed new functions. Gene expression appears to be a useful indicator of their likely roles in secondary growth in vegetative tissues [87]. Hence, we searched the database for expression of the switchgrass representatives of the grass-expanded clades. Figure 9 shows that three out of the nine genes for which data were available show strong expression in stems in general and vascular bundles in particular. Thus, these genes represent potential novel contributors to grass vegetative SCW regulation now under investigation.

## Conclusions

A key element of translating basic research on model (or reference) species, such as Arabidopsis, to crops for food and fuel, is understanding the relative gene complement of the species in question, many of which, like switchgrass, possess a complex genome [64]. We have sought to address this need for the R2R3 MYB proteins. The three tools, phylogenetic analysis, sequence identity, and OrthoMCL analysis, for indicating orthologous relationships that we employed have various requirements for time and expertise. Multi-species phylogenetic analysis appears to be relatively inclusive in its groupings and is informative regarding the rough evolutionary history, such as the occurrence of gene or genome duplication and speciation. However, the topology of a phylogentic tree (1) can be model-dependent, especially for divergent sequences and (2) does not indicate which members of expanded groups are the most similar to those in other species, for example proteins in Class III that have expanded and functionally diverged in Arabidopsis. In addition, phylogenetic analysis is time consuming and, thus, infrequently used for genome-scale analysis.

In contrast to phylogenetic analysis, OrthoMCL, once implemented, can rapidly analyze multiple genomes. A previous comparative analysis of OrthoMCL and other similar large-scale ortholog identification methods found that OrthoMCL and the similar algorithm, InParanoid, have relatively high specificity and sensitivity on a “gold standard” data set [86]. However, in the analysis presented here, OrthoMCL fails to identity known orthologs across dicots and grasses, as for the MYB46/83 and the MYB4/32 clades, though simple sequence identity supports the evidence of functional conservation across dicots and monocots in those clades. This indicates a problem with false negatives, if we select orthologs only based on OrthoMCL. Conversely, sequence similarity groups the grass co-orthologs in the MYB61/50 clade with the Cd^2+^-tolerance regulator, AtMYB50, for which the function is unknown. In that case, the OrthoMCL cluster may be more consistent with the functional data than the sequence identity data. (Alternatively, AtMYB50 may also function in SCW regulation.). For both tools, the quantitation of similarity may not be generally applicable across the genome and lead to false grouping or grouping failure. Ideally, a genome-scale syntenic analysis across species could be an additional piece of information to assist in identifying orthologs when a more accurate and complete switchgrass chromosomal assembly becomes available.

The switchgrass gene expression dataset, when available, appears to provide a much more nuanced guide of function among putative orthologs. For example, expression data suggest that among the switchgrass co-orthologs from the MYB46/83 and MYB42/85 clades, *AP13ISTG55479* and *AP13CTG22878*, are predominantly expressed and potentially better targets for reverse genetics compared with their paralogs. The gaps in the expression dataset provide support for applying and consolidating other transcriptomics approaches, such as RNA Seq [88].

Comparative analysis of the R2R3 MYB family reinforces the assertion that though largely conserved, grass and dicot MYB families have undergone expansions and contractions (Table 3). With respect to SCW regulation, our analysis and emerging functional data [45,76] are largely consistent with general but not complete, conservation of the Arabidopsis regulatory network (Figure 1). Phylogenetic and in some cases, gene expression data, for almost all of the AtMYBs grouped in classes I, II, and III, support conservation. This is despite the ambiguity of the class III proteins, which appear to have undergone expansion and neofunctionalization in the Arabidopsis lineage. This result is consistent with other global analyses of SCW regulation, such as based on maize gene expression data [89]. Among established MYB SCW regulators, the repressor AtMYB75 is clearly not conserved and hence falls in class IV in our analysis. In addition, the MYB20/43 clade gene expression data in switchgrass and the reverse genetic data in Arabidopsis question the inclusion of these proteins among SCW regulators.

Differences between dicot and grass SCW regulation are likely to exist. In support of this, the gene expression data from switchgrass suggest that the expansion of SCW R2R3 MYB proteins, either through whole genome duplication or more specific processes, has led to subfunctionalization in that species. For example, co-orthologs of *AtMYB4* and *AtMYB32*, namely, *AP13ISTG73550*, *AP13ISTG65360*, and *PvMYB4.d*, exhibit not just different expression amounts, but different expression patterns relative to each other (Figure 9). In addition, we identified several grass-expanded R2R3 MYB subgroups and clades (Table 3, Figures 4 and 7) that may possess novel roles in grass-specific biology, including cell wall development. Some of these proteins are highly expressed in stems (Figure 9). Hence, this comparative analysis of the R2R3 MYB family will support the analysis of grass genomic data, providing particular insight into the emerging switchgrass genome. This information can be used to promote biofuel production from switchgrass and other grasses.

## Methods

### Identification of R2R3 MYB proteins

We used HMMER 3.0 [68] to identify the putative R2R3 MYB sequences in different species with an in-house Hidden Markov Model profile based on the 126 R2R3 MYB proteins in Arabidopsis [2]. We mined the following genome annotation versions, which were current at the time of the analysis: *Oryza sativa*, MSU v7; *Populus trichocarpa*, Phytozome v3.0; *Zea mays*, Phytozome v2.0; *Arabidopsis thaliana*, TAIR v10; *Panicum virgatum*, Phytozome v0.0 DOE-JGI, (http://www.phytozome.net/panicumvirgatum), and the unitranscripts dataset from the Switchgrass Functional Genomics Server (http://switchgrassgenomics.noble.org/) [73]. The switchgrass gene identifiers from Phytozome are “Pavirv” and those from the Switchgrass Functional Genomics Server are “AP13” and “Kanl”. Only a few genes in the dataset have multiple known gene models, thus we used only gene model one (.1) for all analyses.

In our initial analysis of the switchgrass R2R3 MYBs in the v0.0 annotation, we noticed that expected sequences, namely, the recently characterized PvMYB4 proteins [45], were missing. A transcript with high homology was present in the v0.0 set of annotated coding sequences, suggesting that the omission was likely during the quality control of the protein annotation. To help to address this, we incorporated the proteins encoded by the unitranscripts in the Switchgrass Functional Genomics Server, which includes Sanger and 454 transcripts from Alamo (AP13) and Kanlow (Kanl) cultivars [73]. To identify switchgrass MYB proteins, we translated the transcripts, which are all the forward strands, using Bioperl, and screened them with the Arabidopsis R2R3 MYB Hidden Markov Model profile. The resulting putative MYB proteins were trimmed to remove the amino acids encoded by the RNA untranslated regions. The numeral (0, 1, 2) appended to the unitranscript sequence identifiers indicates the translation frames of the putative MYB, with “.0” indicating the +1 frame, etc. We compared the unitranscript-derived MYBs and the Phytozome switchgrass v0.0 protein datasets, and deleted the 100% redundant sequences from the Phytozome protein sequences for subsequent analysis. We also included the five sequences of the recently characterized PvMYB4 [45]. Of those, PvMYB4.d is the only sequence that we found in the unitranscript dataset with the sequence identifier *AP13ISTG63786.0*.

We did an initial alignment of the R2R3 MYBs of each species using ClustalW2.0 and then removed sequences that lacked the R2R3 repeats. We also removed sequences that lacked two PROSITE (http://prosite.expasy.org/scanprosite/, PS50090) R repeats [14,72]. The final set of protein sequences and corresponding locus IDs or transcript identifiers used in this analysis is available in Additional file 1: Table S1.

### Phylogenetic and orthoMCL analyses

We used CLUSTALW2.0 for all alignments, which we examined for quality, but did not need to edit. We randomly selected AmMYB6 from *Apis mellifera* as an outgroup. We used MEGA5.0 to infer phylogenetic relationships among the putative R2R3 MYB proteins. For the five species tree, we used the Neighbor-Joining algorithm with the default settings, except that gaps were treated by pair-wise deletion [90]. For the R2R3 MYB multispecies tree we used 500 bootstraps. For each of the SCW regulators, we inferred the relationships with the Maximum-Likelihood algorithm using 1000 bootstraps. The tree topologies were the same between Neighbor-Joining and Maximum-Likelihood algorithms. Within the SCW-related phylogenetic trees, we have identified SCW-protein containing and grass-expanded clades based on bootstrap scores of ≥50 and delimit these with dashed lines. In these trees, we define grass-expanded clades as having more members in rice than in either of the dicots. Most of these clades do not appear to be represented in Arabidopsis or poplar. To further examine homologous relationships among the R2R3 MYB proteins from the five species, we applied OrthoMCL analysis with the default settings [71].

By convention, “homolog” is a general term for proteins that share a common origin and includes both “orthologs” and “paralogs.” Orthologs derive from a single protein in the last common ancestor and tend to maintain similar function. Paralogs, on the other hand, are distinguished by being more similar to other proteins within the same genome and hence generated from expansion subsequent to the last common ancestor. Thus, it is harder to predict the function of paralogs across species, since expansion of the clade may have provided the opportunity for neo- or sub-functionalization [74].

### Sequence identity calculation and allelic diversity

Sequence similarity scores were calculated based on Multiple Sequence Alignment (MUSCLE) with the full-length protein sequences using DNA Subway (http://www.iplantcollaborative.org/discover/dna-subway). Through this analysis, some proteins appeared to have very high protein sequence similarity, consistent with being alleles or splice-variants of the same gene. There is no consensus on the criteria to identify alleles based on nucleotide or protein sequences similarity. Here, we highlight proteins with ≥99% similarity of amino acid sequences as possible alleles or splice-variants.

### Conserved motifs

We analyzed the presence of conserved motifs in the full-length R2R3 MYB proteins from the 48 subgroups (and 4 sub-subgroups) separately with MEME (http://meme.nbcr.net/meme/intro.html) using the following parameters: distribution of motif occurrences: one per sequence and present in all; number of different motifs: 10; minimum motif width: 6; maximum motif width: 15. Identified motifs C-terminal to the MYB domain with E-values lower than 1E-03 are listed in Additional file 3: Table S2. To put our results in the context of the literature, the regular expression of each motif was compared to those previously identified for the Arabidopsis R2R3 MYB family [13].

### Gene expression

We used the gene expression data available from the Switchgrass Functional Genomics Server: http://switchgrassgenomics.noble.org/index.php [73]. The Gene Expression Atlas available through that server was assembled from Affymetrix microarray technology with 122,868 probe sets corresponding to 110,208 *Panicum virgatum* unitranscript sequences to measure gene expression in all major organs at one or more stages of development from germination to flowering [73]. Using heatmap.2 in R, we plotted the log_2_ of the Affymetrix hybridization signals, which represents the normalized mean values of three independent biological replicates for a given organ/stage/tissue. Data are available for only a subset of switchgrass gene models, presumably due to not being represented, at all or uniquely, on the Affymetrix array.

## Availability of supporting data

The data supporting this analysis are available within the Additional files.

## Abbreviations

SCW: Secondary cell wall; R: Repeat; G: Subgroup; At: *Arabidopsis thaliana*; Os: *Oryza sativa*; Pv: *Panicum virgatum*; Ptr: *Poplulus trichocarpa*; Zm: *Zea mays*; SND: Secondary wall-associated NAC domain protein; NST: NAC secondary wall thickening factor; E4: Elongation 4 stage; RNAi: RNA interference.

## Competing interests

The authors declare that they have no competing interests.

## Authors’ contributions

KZ and LEB conceived of and designed the study and wrote the manuscript. KZ carried out the analyses and created the figures. All authors read and approved the final manuscript.

## Supplementary Material

Additional file 1: Table S1 R2R3 MYB protein sequences and names from Arabidopsis, poplar, rice, maize and switchgrass. Click here for file

Additional file 2: Figure S1 Neighbor-joining tree of R2R3 MYB family proteins from Arabidopsis, poplar, rice, maize and switchgrass with 500 bootstraps in .PNG format [88]. Click here for file

Additional file 3: Table S2 C-terminal motif analysis of R2R3 MYB protein in designated subgroups. Click here for file
