# Supplementary figures and images for: Comparative genomic analysis of the R2R3 MYB secondary cell wall regulators of Arabidopsis, poplar, rice, maize, and switchgrass
Source: BMC Plant Biol. 2014 May 18;14:135. doi: 10.1186/1471-2229-14-135 (PMC4057907; doi:10.1186/1471-2229-14-135)

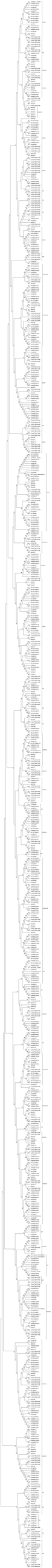

Supplement: Additional file 2: Figure S1 — Neighbor-joining tree of R2R3 MYB family proteins from Arabidopsis, poplar, rice, maize and switchgrass with 500 bootstraps in .PNG format [88]. [file 1471-2229-14-135-S2.png]
